# The Michael addition of thiols to 13-oxo-octadecadienoate (13-oxo-ODE) with implications for LC-MS analysis of glutathione conjugation

**DOI:** 10.1016/j.jbc.2024.107293

**Published:** 2024-04-16

**Authors:** William E. Boeglin, Donald F. Stec, Saori Noguchi, M. Wade Calcutt, Alan R. Brash

**Affiliations:** 1 Department of Pharmacology, Vanderbilt University, Nashville, Tennessee, USA; 2 Vanderbilt Institute of Chemical Biology, Vanderbilt University, Nashville, Tennessee, USA; 3 Department of Chemistry, Vanderbilt University, Nashville, Tennessee, USA; 4 Department of Biochemistry, Vanderbilt University, Nashville, Tennessee, USA

**Keywords:** keto fatty acid, oxo fatty acid, HPLC, mass spectrometry, ^1^H-NMR, UV-Vis spectrometry

## Abstract

Unsaturated fatty acid ketones with αβ,γδ conjugation are susceptible to Michael addition of thiols, with unresolved issues on the site of adduction and precise structures of the conjugates. Herein we reacted 13-keto-octadecadienoic acid (13-oxo-ODE or 13-KODE) with glutathione (GSH), N-acetyl-cysteine, and β-mercaptoethanol and identified the adducts. HPLC-UV analyses indicated none of the products exhibit a conjugated enone UV chromophore, a result that conflicts with the literature and is relevant to the mass spectral interpretation of 1,4 *versus* 1,6 thiol adduction. Aided by the development of an HPLC solvent system that separates the GSH diastereomers and thus avoids overlap of signals in proton NMR experiments, we established the two major conjugates are formed by 1,6 addition of GSH at the 9-carbon of 13-oxo-ODE with the remaining double bond α to the thiol in the 10,11 position. N-acetyl cysteine reacts similarly, while β-mercaptoethanol gives equal amounts of 1,4 and 1,6 addition products. Equine glutathione transferase catalyzed 1,6 addition of GSH to the two major diastereomers in 44:56 proportions. LC-MS in positive ion mode gives a product ion interpreted before as evidence of 1,4-thiol adduction, whereas here we find this ion using the authentic 1,6 adduct. LC-MS with negative ion APCI gave a fragment selective for 1,4 adduction. These results clarify the structures of thiol conjugates of a prototypical unsaturated keto-fatty acid and have relevance to the application of LC-MS for the structural analysis of keto-fatty acid glutathione conjugation.

Unsaturated keto fatty acids arise from polyunsaturated precursors *via* enzymic or non-enzymic pathways (*e.g.* refs (1, 2, 3, 4, 5)). Enzymic routes go through the initial formation of a hydroxy fatty acid, followed by dehydrogenase-catalyzed oxidation to an αβ,γδ-unsaturated ketone (*e.g.* refs (6, 7, 8, 9, 10)). For example, this accounts for the production of 13-oxo-9*Z*,11*E*-octadecadienoic acid (13-oxo-ODE or 13-KODE) *via* 13-hydroxy-octadecadienoic acid in rat tissues, with notable high activity in liver and colon (6, 11). Arthur Bull and coworkers went on to establish a role for 13-oxo-ODE in intestinal cell differentiation (12) and, in pursuing the mechanism, their finding of protein adduction by 13-oxo-ODE (13). Analysis of reaction rates with individual amino acids demonstrated relatively high rates of adduction (albeit requiring high pH) only with cysteine (13). There followed an HPLC and NMR study of the non-enzymic Michael adduction of 13-oxo-ODE with N-acetyl-cysteine and glutathione (GSH) (14). This and subsequent studies by the same group demonstrated that the two diastereomers produced by adduction with glutathione are formed in 1:1 proportion non-enzymatically and in varied proportions depending on the GST enzyme employed (14, 15, 16).

In the course of our own development of methods for the purification and NMR analysis of cysteinyl adducts of linoleate epoxy-ketones, we used the reported reactions of 13-oxo-ODE as a test case. In doing so, our initial HPLC runs with diode array UV detection showed that the GSH-13-oxo-ODE adducts lack any conjugated UV chromophore, which is at odds with the structure deduced in the earlier work, Figure 1, *A* and *B*. Furthermore, several studies after the reported 13-oxo-ODE adduction concluded by LC-MS analysis that thiol adduction to the particular fatty acid in question occurs in the middle of the conjugated dienone of the unsaturated keto substrate (4, 17, 18, 19, 20, 21), Figure 1*C*. Thus, the thiol adduction of 13-oxo-ODE is of relevance to a body of work on glutathione conjugation of αβ,γδ-unsaturated keto fatty acids (22, 23, 24).

**Figure 1:**
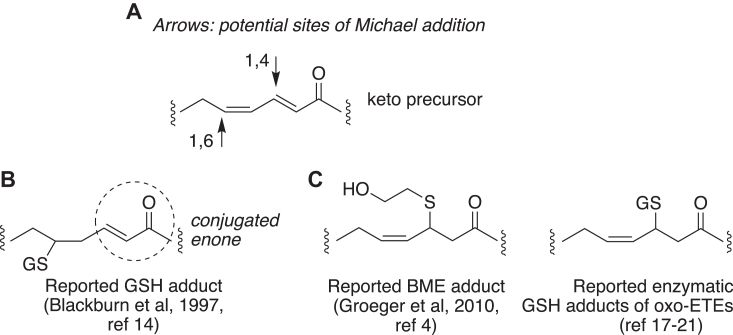
**An αβ,γδ-unsaturated fatty acid ketone and reported GSH adducts.***A*, potential sites of Michael addition of thiols to an αβ,γδ-unsaturated fatty acid ketone, (*B*) reported 13-oxo-ODE adduct with glutathione (14), which includes a conjugated enone UV chromophore in the structure, and (*C*) adducts with other αβ,γδ-unsaturated keto fatty acids, either with BME (4), or oxo-ETEs (oxo-eicosatetraenoic acids) (17, 19, 20, 21).

In purifying and preparing 13-oxo-ODE-GSH for NMR analysis, we encountered two technical hurdles that were partly solved or circumvented in the present study. Two issues related to the HPLC purification of the conjugates, particularly pertained to the polar amino acid adducts. On conventional HPLC solvent systems, the two diastereomeric adducts were not resolved from one another, and quite modest amounts of adducts (anything over 5 μg injected on a 0.46 cm diameter analytical column) produced early elution with progressively broadening peaks as higher amounts were injected. This further confounded the purification of diastereomeric adducts. Second, given the limited amounts of samples that could be chromatographically resolved and purified, the quality of the NMR spectra tended to be degraded with interference by relatively large signals from solvents. We finally identified a reversed-phase HPLC solvent that dealt with the early elution/peak broadening issue and also succeeded in separating the two major GSH conjugate isomers and additional side products. Herein we correct the reported structure of the 13-oxo-ODE-GSH adducts, we extend this to the adducts of N-acetyl cysteine, and we find that β-mercaptoethanol (BME) forms adducts equally on two positions of the αβ,γδ-unsaturated ketone. LC-MS analysis of the purified 13-oxo-ODE-GSH conjugates counters and corrects earlier work on the structural interpretation of a key fragmentation, rendering the results directly relevant to the application of LC-MS for the structural analysis of keto-fatty acid glutathione conjugation.

## Results

### UV monitoring of rates of reaction

UV spectroscopy is ideal for monitoring rates of reaction of 13-oxo-ODE *via* the disappearance of the conjugated dienone chromophore (ε 25,000 M^−1^ cm^−1^, λmax 278 nm in methanol, 284 nm in aqueous solution). As reported by Bull and coworkers (13), the non-enzymic adduction of 13-oxo-ODE with cysteine at physiological pH is remarkably sluggish (3–4% of the rate at pH 9). Over the course of a few minutes, the rate is only detectable by UV monitoring at the higher pH values that produce the reactive thiolate anion. Using this approach, reactions were conducted in a 3 ml UV cuvette at pH 8.5 or pH 10 with 20 to 30 μg/ml 13-oxo-ODE and an excess of thiol, either glutathione, N-acetyl-cysteine, or β-mercaptoethanol. Preparative scale reactions used 100 μg/ml 13-oxo-ODE, which saturates the UV signal although still useful for monitoring reductions in the entire UV chromophore.

### HPLC purification of adducts

**Figure 2:**
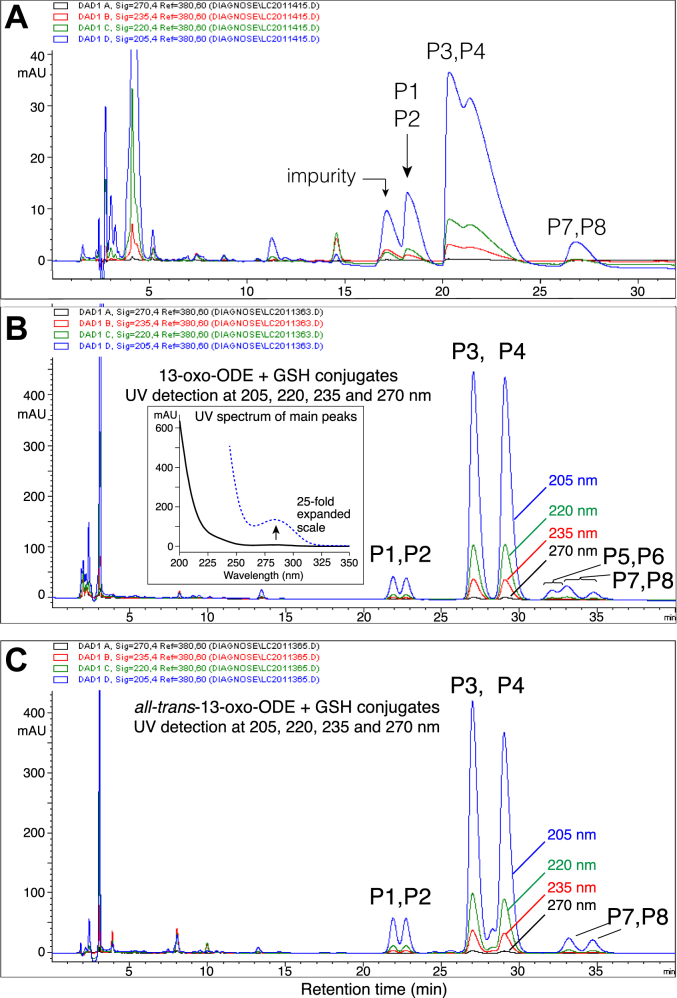
**RP-HPLC of glutathione adducts with 13-oxo-ODE and *all-trans*-13-oxo-ODE.** The reaction products of GSH were analyzed with isocratic elution on a Waters C18 5 μ Symmetry column (25 × 0.46 cm) at a flow rate of 1 ml/min and with UV detection at 205 nm (*blue*), 220 nm (*green*), 235 nm (*red*) and 270 nm (*black*). *A*, an example of the RP-HPLC analysis of *all-trans*-13-oxo-ODE-GSH conjugates using a conventional solvent system of acetonitrile/water/glacial acetic acid (65:35:0.01 by volume). *B* and *C*, the reaction products of GSH with (*B*) 13-oxo-ODE or (*C*) *all-trans*-13-oxo-ODE were analyzed using a solvent system of 30:70 acetonitrile/50 mM KH_2_PO_4_ (adjusted with H_3_PO_4_ to pH 2). The inset in *panel B* shows the UV spectrum of the main peaks designated as P3 and P4; at 25 times higher sensitivity, the weak ∼285 nm absorbance of an isolated ketone is detectable. The small peaks on either side of the main peaks (P1, P2, P5-P8) have similar UV spectra.

Using conventional acetonitrile/water/acetic acid solvents for RP-HPLC of the 13-oxo-ODE-GSH conjugates gave exceptionally broad peaks and poorly resolved mixtures of isomers, Figure 2*A*. Separation of the products of GSH conjugation with 13-oxo-ODE and *all-trans*-13-oxo-ODE was achieved using an RP-HPLC solvent system with an aqueous component of 50 mM potassium phosphate adjusted to pH 2 with phosphoric acid, Figure 2, *B* and *C*. Not only does this buffer avoid problems with peak broadening and early elution when 50 to 100 μg amounts are injected into the column, but unlike the other solvents we tested, it nicely resolves the main 13-oxo-ODE-GSH diastereomers. The contrast in HPLC profiles using acetic acid *versus* the pH 2 phosphate as the aqueous component of the solvent is illustrated for consecutive injections on column in Fig. S1.

Notably, the reaction of *all trans*-13-oxo-ODE (9*E*,11*E*) with GSH produced the same HPLC profile as for the *cis-trans*-13-oxo-ODE (9*Z*,11*E*), Figure 2, *B* and *C*. If thiol adduction occurs at C9 (as the NMR analyses below establish) is not unexpected that the *cis-trans* and *trans-trans* 13-oxo-ODE isomers produce the same major diastereomers. Furthermore, *cis-trans* 13-oxo-ODE is isomerized during reaction with thiols, as evidenced by the unreacted substrate being recovered mainly as *all-trans* 13-oxo-ODE (Fig. S2).

**Figure 3:**
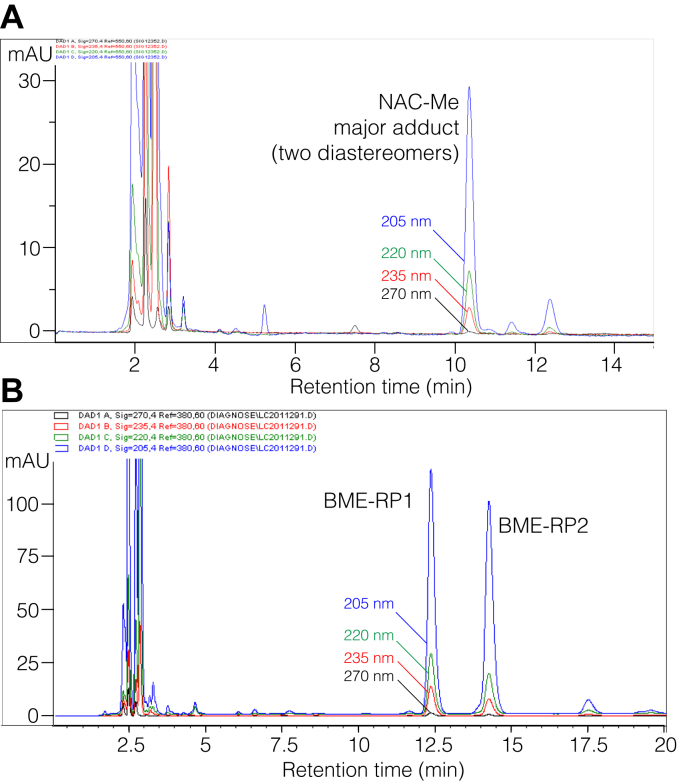
**RP-HPLC of the NAC and BME adducts of 13-oxo-ODE.** Analytical separation of the adducts of (*A*) NAC methyl ester and (*B*) 13-oxo-ODE-BME adducts using a Waters C18 5 μ Symmetry column (15 × 0.2 cm) and an isocratic solvent of acetonitrile/water/glacial acetic acid (60:40:0.01 by volume) at a flow rate of 0.3 ml/min and with UV detection at 205 nm (*blue*), 220 nm (*green*), 235 nm (*red*) and 270 nm (*black*).

With less polar conjugates, chromatographic behavior was normal, and 100 μg quantities could be run with minimal peak broadening or early elution. This applied to the conjugates of 13-oxo-ODE methyl ester with N-acetyl-cysteine methyl ester or BME, Figure 3, *A* and *B*. Both were also readily amenable to normal-phase HPLC (Experimental procedures), providing an additional measure of purification and helping to eliminate traces of polar solvents prior to NMR.

### HPLC-UV of adducts

On HPLC with diode array detection, the UV spectra of the glutathione and other thiol adducts with 13-oxo-ODE showed no conjugated chromophore, only end-absorbance near 200 nm with minor inflections of the absorbance as the signal reduces down to near zero by 250 nm. (The spectrum of the major GSH-13-oxo-ODE adducts is illustrated in the inset of Fig. 2). This observation provided the first clear evidence that adduction completely eliminates conjugation of the 13-ketone with a double bond. In other words, none of the adducts include a sub-structure with the 13-ketone conjugated to an 11,12 double bond as originally deduced for the glutathione and N-acetyl-cysteine adducts (14).

### Proton NMR of 13-oxo-ODE-glutathione adducts

**Figure 4:**
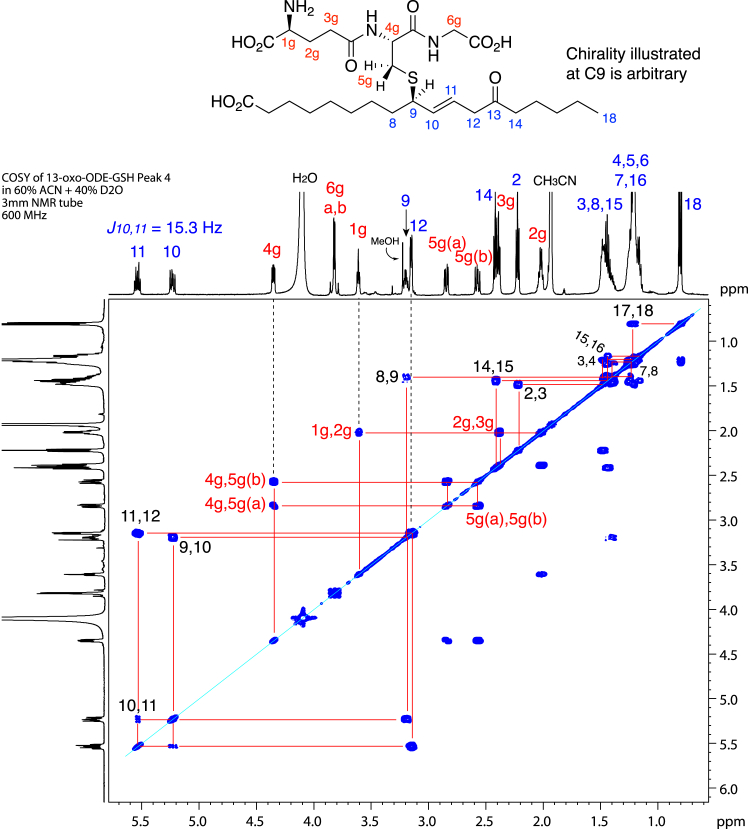
^**1**^**H-NMR spectrum and COSY analysis of P4, a major 1,6 adduct of 13-oxo-ODE-GSH (600 MHz, in CD**_**3**_**CN/D**_**2**_**O, 60:40 by volume).** The sample was run on a Waters C18 5 μ Symmetry column (25 × 0.46 cm) and an isocratic solvent of acetonitrile/water/glacial acetic acid (70:30:0.01 by volume) at a flow rate of 1 ml/min and with UV detection at 205 nm (illustrated), 220 nm, 235 nm and 270 nm.

The 1D spectra with 2D COSY analyses of the two major 13-oxo-ODE-GSH diastereomers establish the structures as thiol conjugates of GSH at C9 of 13-oxo-ODE with the one remaining double bond α to the thiol at 10,11 (Figs. 4, S3 and S4 and Supporting Information Text). Establishing the position of the double bond impacts the reported applications of LC-MS for structural analysis of oxo fatty acid glutathione conjugates (17, 18, 19, 20, 21), and is considered later in detail, *vide infra*.

Key interpretations of the ^1^H-NMR spectra, taking Figure 4 and the product P4 as the example, can start with the CH_2_ at 3.25 ppm (H12). H12 is a doublet, α to the 13-ketone, and from the COSY, its single proton neighbor is on the double bond at 5.64 ppm (H11), itself coupled to H10 at 5.38 ppm, which couples to the single proton at 3.33 ppm (C9, the site of thiol adduction), with its coupling to the upfield C8 protons at 1.52 ppm. The identical set of couplings is evident on the second major isomer (P3, Figs. S3 and S4 and Supporting Information Text). Also seen in these spectra are the protons from glutathione, labeled in red in Figure 4 and on the accompanying chemical structure. The glutathionyl proton chemical shifts are in accord with the ^1^H-NMR spectrum of glutathione itself (illustrated in the Supplement of ref (25)).

The minor products of the 13-oxo-ODE/GSH transformation in Figure 2 were purified and analyzed separately by negative ion ESI-LC-MS and found to all share the same M-1 ion of a 13-oxo-ODE-GSH conjugate, m/z 600.2946 (calc. 600.2960). ^1^H-NMR spectra and COSY analyses were obtained on representative peaks P1 and P8 from Figure 2. The results established conclusively that P1, and thus its diastereomer P2, are conjugates of GSH at C9, differing from the major products in having a 10,11-*cis* double bond (Fig. S5 and Supporting Information Text). NMR analyses prove P8 is a 1,4-addition product with GSH adducted to C11 of 13-oxo-ODE, with a remaining 9,10-*trans* double bond (Fig. S6 and Supporting Information Text). P7 is the diastereomer of P8 (Fig. 2*B*), leaving the conclusion on multiple lines of evidence that P5 and P6 are the 1,4-addition products retaining the 9,10-*cis* double bond (Supporting Information Text).

### Proton NMR 13-oxo-ODE-methyl ester adducts with N-acetyl-cysteine methyl ester

To circumvent issues with purification of adducts and final removal of polar solvents for NMR analysis, the methyl ester of 13-oxo-ODE was reacted with the methyl ester of N-acetyl cysteine; the reduced polarity of the methyl esters allowed for both RP-HPLC and NP-HPLC purification of the adducts (which co-chromatograph is both HPLC systems), and also permitted their solubility in d_6_-benzene for NMR analysis. Fortunately, with the aid of COSY analysis, the proton NMR spectra of the two diastereomers could be distinguished from one another, Fig. S7. In both isomers the NAC-Me group is adducted at C-9, as evidenced by its coupling to the more upfield of the two double bond protons (H10 at 5.23 ppm), then *via* the second double bond proton (H11 at 5.55/5.63 ppm in the two isomers), their coupling to the doublet of H12 at 2.78 ppm confirms the location of the 10,11-*trans* double bond (*J*_*10,11*_ = 15.2 Hz), the same as in the GSH conjugates.

### Proton NMR of 13-oxo-ODE methyl ester-β-mercaptoethanol adducts

**Figure 5:**
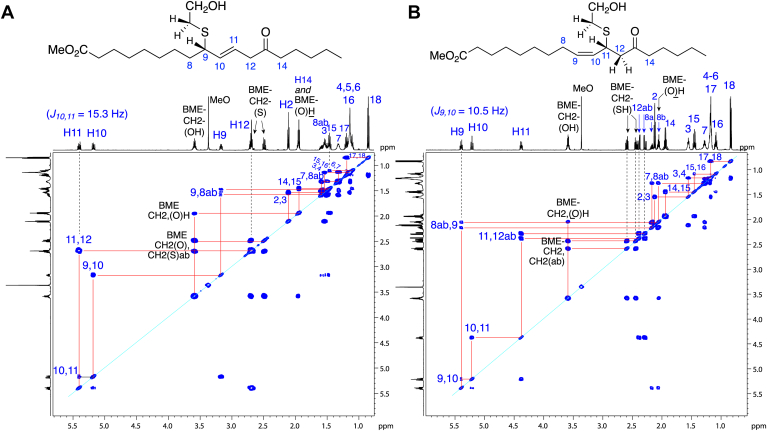
^**1**^**H-NMR spectrum and COSY analysis of conjugates of BME with 13-oxo-ODE.** The samples were methylated with diazomethane after conjugation, purified as in Figure 3, and the NMR recorded in d_6_-benzene. *A*, the more polar product RP1-NP2, a 1,6 addition product conjugate at C9. *B*, the less polar product, RP2-NP1, a 1,4 addition product at C11 of 13-oxo-ODE.

Adduction with BME has been applied for the LC-MS quantitative analysis of αβ.γδ-keto fatty acid production in cells (4), representing a practical relevance for structural analysis of the conjugates. Following the reaction of 13-oxo-ODE with BME, the extract was methylated with diazomethane, and the conjugates were separated by RP-HPLC (Fig. 3*B*) and further purified on NP-HPLC. In order of elution, the two equal-sized peaks were designated RP1-NP2 (the more polar) and RP2-NP1 (less polar). Their ^1^H-NMR spectra were recorded in d_6_-benzene, Figure 5, *A* and *B*.

The more polar product RP1-NP2 has 1,6 adduction of the thiol to C9 of 13-oxo-ODE (Fig. 5*A*). Although the signal from H12 next to the ketone overlaps with one of the CH_2_ protons in BME, the cross peaks on the COSY clearly demonstrate H12 coupling to the H11 proton on the double bond. From there the coupling to H10 leads back to the geminal proton with the thiol adduct on the 9-carbon. A similar analysis of the less polar product RP2-NP1 in Figure 5*B* shows clear signals indicating 1,4 adduction of BME at C11 of 13-oxo-ODE (detailed in Supporting Information Text).

### Equine GST adduction of GSH to 13-oxo-ODE

**Figure 6:**
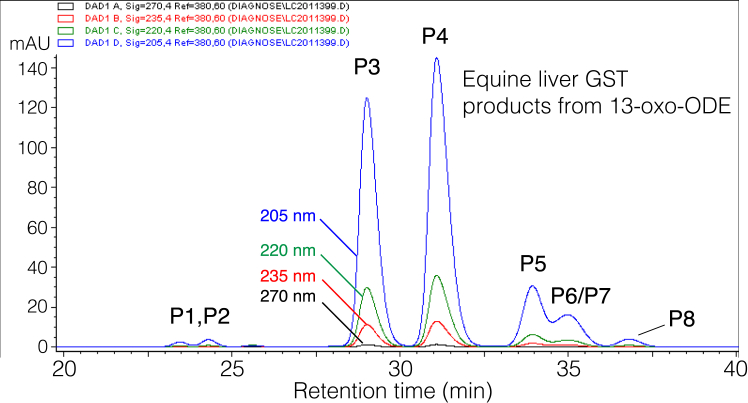
**RP-HPLC of glutathione adducts with 13-oxo-ODE catalyzed by equine GST.** The chromatography (showing 20–40 min retention time) used the same column and solvents as in Figure 2, *B* and *C* with UV detection at 205 nm (*blue*), 220 nm (*green*), 235 nm (*red*), and 270 nm (*black*).

Equine glutathione transferase (GST) was used to establish the adduct structures formed in a prototypical enzymic transformation of GSH with 13-oxo-ODE. The equine liver GST catalyzed efficient adduction at pH 7.5, a pH value with minimal non-enzymic transformation during the 1-h time course. The major products were formed by 1,6 addition to C9 of 13-oxo-ODE in 44:56 proportion, Figure 6. In comparison to the reaction profile of the non-enzymic addition, the early peaks were smaller (P1, P2, the 1,6 adducts with a 10,11-*cis* double bond), and there is a slightly larger peak of the first 1,4 adduct (P5, Fig. 6).

### LC-MS-ESI analysis of the 13-oxo-ODE-GSH adducts

**Figure 7:**
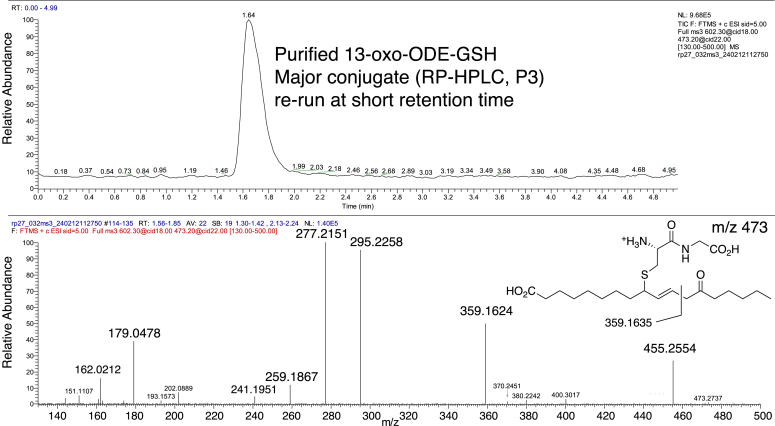
**LC-MS-ESI in positive ion mode with MS**^**3**^**on 1,6-adduct of 13-oxo-ODE-GSH.** The spectrum shown is from fragmentation of m/z 602 → 473 → spectrum including m/z 359.

Five previous publications used positive ion mass spectrometry for the structural analysis of GSH conjugates of αβ,γδ-unsaturated fatty acid ketones, and all concluded that the mass spectral fragmentation provided evidence of 1,4-adduction of GSH (17, 19, 20, 21, 26). Here we examined the evidence using the purified and authentic 1,4- and 1,6-adducts. The 13-oxo-ODE-GSH conjugates in Figure 2 were collected separately, re-extracted on an Oasis cartridge to remove the potassium phosphate buffer, then one of the major diastereomers and representative minor products eluting before and after the main peaks on RP-HPLC were analyzed by ESI in the positive ion mode. Using the purified 1,6 adduct (P3 from Fig. 2) with exact mass analysis and MS^3^ (m/z 602 → 473 → 359), the results counter the basis of the earlier conclusions by demonstrating the formation of m/z 359 representing C1–C11 of the fatty acid with loss of the glutamyl amino acid from the GSH tripeptide, Figure 7. The m/z 359 ion was used as evidence of 1,4 adduction of 13-oxo-ODE-GSH (26), and the equivalent fragmentation was implicated in other fatty acid unsaturated ketone conjugates (17, 19, 20, 21). Extending our MS/MS analyses to the minor 13-oxo-ODE conjugates showed that all produce an m/z 359 ion in MS^3^ positive ion analysis, albeit in low relative abundance, Fig. S8.

### Negative ion APCI of the 13-oxo-ODE-GSH adducts

**Figure 8:**
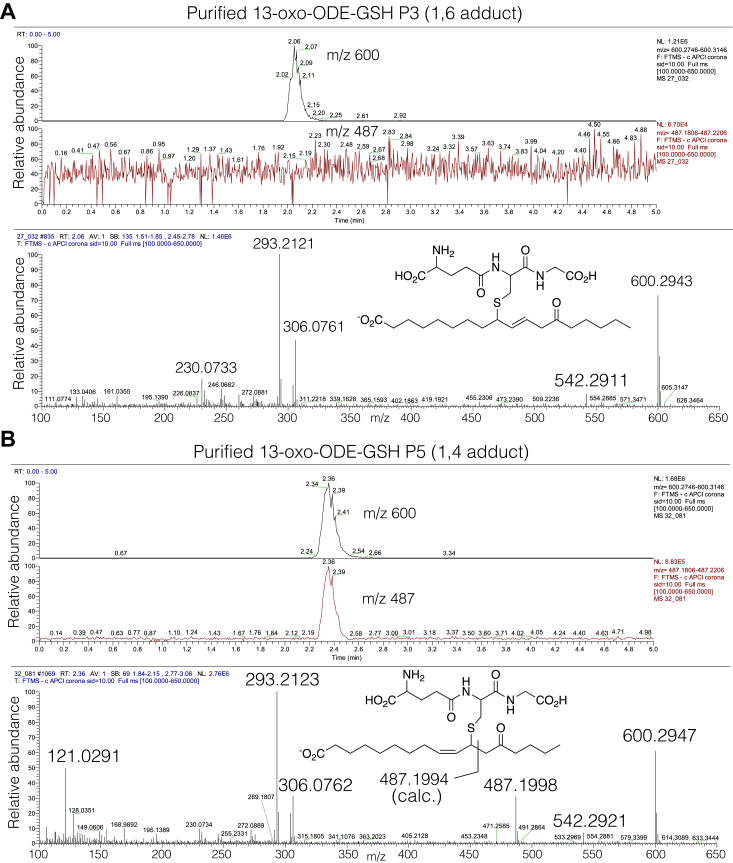
**LC-MS in negative ion APCI of representative 1,4 and 1,6 adducts of 13-oxo-ODE-GSH.***A*, negative ion APCI mass spectrum of the 1,6-adduct of 13-oxo-ODE-GSH, product P3 from Figure 2. The selected ion monitoring for P3 show the profile for M-H^-^ ion m/z 600, and no detectable m/z 487. *B*, by contrast, the mass spectrum of the 1,4-adduct P5 from Figure 2 shows a strong m/z 487 ion representing C1-C11 of the fatty acid chain and including the GSH tripeptide, and the selected ion monitoring profiles confirm co-chromatography of m/z 600 and m/z 487.

As an alternative to the positive ion ESI analyses, the purified 13-oxo-ODE-GSH conjugates were analyzed by negative ion APCI, and the method appears promising for distinguishing 1,4- and 1,6-adduction. Spectra were obtained on representative conjugates (purified as in Fig. 2). P5, P6, and P8, representing 1,4-addition, give a prominent cleavage ion, m/z 487, containing the glutathione tripeptide and C1 – C11 of 13-oxo-ODE. This ion is undetectable in the representative 1,6 addition products P1 and P3. Figure 8 illustrates the results for the major 1,6 adduct P3 and for the first of the 1,4 adducts, P5, and the spectra of P1, P6, and P8 are in Fig. S8.

## Discussion

### Structures of the thiol adducts

**Figure 9:**
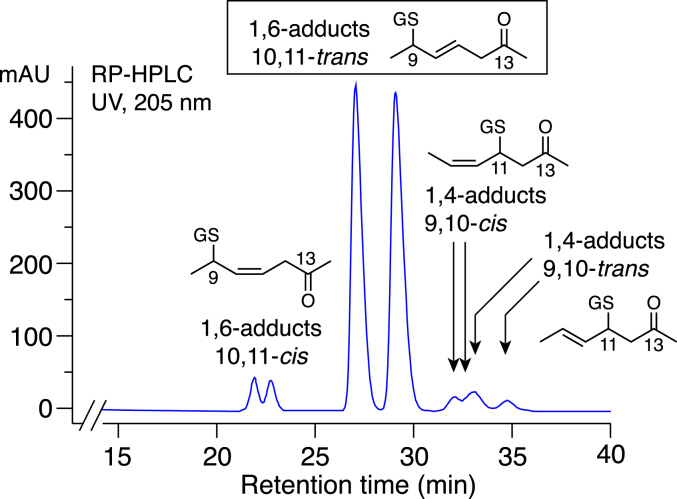
**Summary of the structures of 13-oxo-ODE-GSH conjugates from**Figure 2**.** Partial structures are shown for P1 – P8 from Figure 2.

Figure 9 summarizes the partial structures and relative abundance of the non-enzymatically formed 13-oxo-GSH conjugates in relation to their appearance on RP-HPLC. The results establish the major site of adduction of glutathione and N-acetyl-cysteine at C9 of 13-oxo-ODE. Our HPLC-UV and NMR analyses also clearly established the position of the remaining double bond, which was misplaced in the original study (14). With the adduction of GSH on the 9-carbon, the double bond moves out of conjugation with the 13-ketone to the 10,11 position. While the NMR spectra are definitive on this point, the deduction is also strongly supported by the UV spectra obtained online with diode array detection; none of the 13-oxo-ODE adducts of GSH, NAC or BME in the present study exhibited a conjugated UV chromophore, not even the minor peaks at the side of the major products. The position of the double bond after 1,6-addition of GSH to 13-oxo-ODE corresponds to literature precedent with thiol adduction to the αβ-γδ-unsaturated sorbic acid; thiols react with this 2,4-dienoic acid with 1,6 addition to C5 with the remaining double bond in the 3,4 position (27, 28). With NAC methyl ester, the major diastereomers were also 1,6 addition products at C9 of 13-oxo-ODE (Figs. 3*A* and S7), while BME adducted about equally at the 9- and 11-carbons (Figs. 3B and 5).

### GST-catalyzed GSH adduction to unsaturated keto fatty acids

In earlier work, the two major 13-oxo-ODE-GSH diastereomers were successfully resolved using a chiral HPLC column (14), and this method was used in a series of papers by Bull and colleagues to demonstrate the selective synthesis of individual adduct isomers in different cell lines and by different glutathione transferases (14, 15, 16). Then, as of now, the R/S stereochemistry at C9 of the isomeric structures of the 13-oxo-ODE-GSH conjugates is not solved and they were designated isomer-1 and isomer-2 in order of elution. HT-29 cells and rat liver GST formed different diastereomers (15), and selective production of one or other isomer was demonstrated by a panel of purified GSTs (16). The adducts were about 90% resolved from each other on the chiral column detected by UV absorbance at 235 nm and identified by retention time (14); whether there might be new isomers formed by the enzymes was not addressed. Using equine liver GST in our study, the adducts on RP-HPLC are recognizable from the non-enzymic profile and mainly feature the two C9 conjugates in unequal proportion (Fig. 7).

### LC-MS analysis of GSH adduction

The enzymic adduction of GSH to αβ,γδ-unsaturated fatty acid ketones is reported for the direct arachidonic acid analogue of 13-oxo-ODE, namely 15-oxo-ETE (15-oxo-eicosatetraenoic acid) (19), as well as for 5-oxo-ETE, 11-oxo-ETE, and 12-oxo-ETE (17, 20, 21). In all four instances, the structure or tentative structure of the conjugate was deduced by mass spectrometry in the positive ion mode, and assigned as 1,4 addition of GSH in the middle of the conjugated dienone system, that is, at C7 of 5-oxo-ETE, C10 of 12-oxo-ETE, C13 of 11-oxo-ETE, and C13 of 15-oxo-ETE. This contrasts with the structure established here for the major non-enzymic and equine GST-catalyzed adducts of 13-oxo-ODE. The GSH conjugate of 5-oxo-ETE, characterized as a 1,4 addition product on the 7-carbon, and given the acronym FOG_7_ (five-oxo-glutathione C7 adduct), was found to be highly potent in stimulating eosinophil as well as neutrophil chemotaxis, and its chemical structure is fundamental to its biological activity.

Adding to the developing questions are the results of a study by mass spectrometry of glutathione adducts of plant αβ,γδ-unsaturated ketones, including the adducts with 13-oxo-ODE (26). The synthetic standards in the study were prepared as described in the 1997 NMR report (14), the same method we used in the current study. By direct infusion of the synthetic conjugates, and employing positive ion ESI and product ion spectra of the m/z 473 ion (loss of the glutamyl residue of the tripeptide adduct), the authors deduced from the appearance of a m/z 359 product ion (C1-C11 of the fatty acid along with the two remaining peptides) that this indicates 1,4 adduction of the thiol to C11 of 13-KODE (26). The authors concluded that 1,6 adduction if it occurred, was not detectable from the ESI product ions (26).

**Figure 10:**
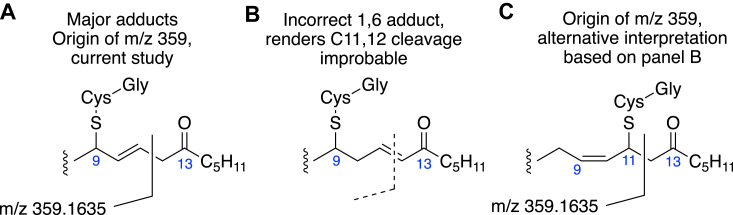
**Potential consequences of correct and incorrect structural assignment of the 1,6-adducts of 13-oxo-ODE-GSH.***A*, as established herein, in positive ion ESI the 1,6-adduct cleaves between C11-C12 to produce an ion at m/z 359. *B*, the incorrect structural assignment with a C11-C12 double bond (14) renders the formation of the m/z 359 ion improbable from the 1,6-adduct. *C*, based on the incorrect assumption in *B*, the m/z 359 ion could be attributed to the C11-C12 cleavage of the 1,4-adduct. As established herein, both the 1,4- and 1,6-adducts fragment in positive ion ESI give a detectable m/z 359 ion, so it is not diagnostic for the placement of GSH adduction.

In retrospect, it seems possible that the analysis of ionization and fragmentation may have been misled by the incorrect structure reported for the 1,6 adduct with the double bond placed in conjugation with the 13-ketone at the 11,12-position (14), Figure 10. As we proved herein, with the double bond in the correct position α to the C9 thiol, cleavage of the 12,13 single bond in the 1,6 adduct produces the ion containing C1-C12 (m/z 359), Figure 10*A*. With the incorrect structure, the equivalent fragmentation across an 11,12 double bond is improbable (Fig. 10*B*), leading to the deduction of GSH conjugation *via* 1,4 addition and accounting for the observed m/z 359 ion fragment (Fig. 10*C*). In fact, our results with the purified 1,4- and 1,6-GSH conjugates of 13-oxo-ODE show that both produce an m/z 359 ion by cleavage through C11-C12.

In the case of the equine GST transformation we examined, the major conjugates are formed *via* 1,6 addition (Fig. 6); however, the correct structures remain an open issue with other substrates and in other systems. For the 13-oxo-ODE-GSH conjugates, the application of negative ion APCI gave a product ion (m/z 487) solely from the 1,4-adducts, although there was no corresponding ion diagnostic for 1,6-adduction. Given the small amounts of conjugates recovered from cultured cells and the ambiguity inherent in the LC-MS analyses our findings expose, comparison with synthetic and NMR-characterized conjugates may be the most secure approach to the identification of the GST-catalyzed adduction of GSH to unsaturated fatty acid ketones.

### Conclusion and biological significance

Adduction with glutathione is known, in general, as a detoxification pathway and for the elimination of drugs and other xenobiotics (22, 24). Multiple families of GSTs are dedicated to this function (29, 30). In the case of 13-oxo-ODE, coupling with GSH has a role in its inactivation and subsequent transport out of the cell (15), as similarly established for its arachidonate analog 15-oxo-ETE (31). A major exception to this role in inactivation is the bronchoconstrictor and other biological activities of the GSH conjugate leukotriene C_4_ and its related peptidyl-leukotrienes (32, 33, 34). Biological activity is also a feature of FOG_7_, the GSH conjugate of the αβ,γδ-unsaturated 5-oxo-ETE, which shares the same molecular formula and molecular weight with LTC_4_ and is highly active as a chemotactic agent for both human eosinophils and neutrophils (17, 23). So far the properties of bioactive FOG_7_ have not been compared with a synthetic standard and its chemical structure is deduced by mass spectrometry. Our study provides the tools to prepare, purify, and analyze the structure of the thiol adducts of any αβ,γδ-unsaturated fatty acid ketone and could be applied to confirm the structure of FOG_7_. Other relevant targets for analysis include each of the partially characterized oxo-ETE-GSH conjugates and their analogs from other polyunsaturated fatty acids, and any of the multiple conjugated enones subject to Michael addition with thiols (35).

## Experimental procedures

### Preparation of 13-oxo-ODE (13-KODE)

The keto acid was prepared by oxidation of 13*S*-HODE using 2,3-dichloro-5,6-dicyano-1,4-benzoquinone (DDQ) (36). 13*S*-HODE was synthesized in 100 mg quantities using soybean lipoxygenase (Sigma, type 5) as described (37). In a typical preparation of 13-oxo-ODE, to 13S-HODE (30 mg, 0.1 μmole) in 0.5 ml dichloromethane in a 5 ml Reactivial on ice, was added one equivalent of solid DDQ (22 mg). The clear solution immediately turned cloudy orange-brown. The sample was loaded onto a 1 g Bond-Elut silica cartridge including a 2 ml rinse of the vial with dichloromethane. The cartridge was eluted with dichloromethane/MeOH/glacial acetic acid (100:1:0.1 by volume) and the 13-oxo-ODE product was collected in three 3 ml fractions that were assayed by UV spectrometry. The DDQ reaction usually resulted in ∼5 to 10% isomerization of the 9*Z*,11*E* bonds to all-*trans* (9*E*,11*E*). The 13-oxo-ODE was purified and the all-*trans* 13-oxo-ODE side-product was removed by NP-HPLC using a semi-preparative silica HPLC column (Thomson Advantage silica, 5 μ, 25 × 1 cm) and a solvent of hexane/IPA/glacial acetic acid 100:2:0.02 (by volume) at a flow rate of 2.5 ml/min (retention volumes 24 ml and 29 ml (13-oxo-ODE and 9*E*,11*E*-13-oxo-ODE). The final yield of 13-oxo-ODE from 13-HODE was 50 to 60%. If further purification was required, 300 μg aliquots of the two products were resolved on an analytical silica column (25 × 0.46 cm), and quantified by UV spectroscopy (ε = 25,000, λmax 279 nm for 13-oxo-ODE and 276 nm for the all-*trans* isomer). For the preparation of 13-oxo-ODE methyl ester, to minimize diazomethane adduction to the unsaturated ketone (*cf.* (38)), the reaction was conducted on ice, with only a few seconds of treatment with diazomethane in 10:1 ether/methanol followed immediately by evaporation under a strong stream of nitrogen. The methyl ester was purified using the semi-preparative silica column and a solvent of hexane/IPA (100:1, by volume).

### Adduction of 13-oxo-ODE and 13-oxo-ODE methyl ester with thiols

Reaction with 25 to 100 μg/ml 13-oxo-ODE was conducted in 0.1 M potassium phosphate (pH 8.5) or 0.1 M borate buffer at pH 10 as reported by Blackburn *et al* (14) and monitored by UV spectroscopy (disappearance of the conjugated dienone chromophore) by repetitive scanning over 350–200 nm using a Cary 60 UV-Vis spectrometer (Agilent). For extraction, the solutions were acidified to pH 3 and applied to a pre-equilibrated 30 mg (for 1–2 ml volumes) or 60 mg Oasis cartridge (up to 10 ml). After loading and washing the cartridge with water, products were eluted with 1:1 acetonitrile/water or methanol.

### Glutathione transferase-catalyzed adduction of 13-oxo-ODE

Equine GST from Sigma was reacted with GSH and 13-oxo-ODE in 0.1 M potassium phosphate pH 7.5 and the transformation was monitored by UV-Vis. Preparative reactions in 3 ml used 100 μg/ml of 13-oxo-ODE, 5 mM GSH, with 100 ng/ml enzyme for 1 h at room temperature. Products were acidified as above and extracted on a 60 mg Oasis cartridge, which was washed with water and the products eluted with 0.75 ml 70:30 acetonitrile/water by volume.

### HPLC-UV analyses

Glutathione conjugates of 13-oxo-ODE were analyzed by RP-HPLC using a Waters Symmetry 5 μm C18 column (25 × 0.46 cm), with an isocratic solvent of acetonitrile/50 mM potassium phosphate adjusted to pH 2 (typically in the proportions 30:70 by volume), although adjusted for the particular sample, at a flow rate of 1 ml/min, with on-line UV detection at 205 nm, 220 nm, 235 nm, and 270 nm (Agilent 1100 series diode array detector). The pH 2 potassium phosphate component of the RP-HPLC solvent is prepared using 50 mM KH_2_PO_4_ (3.4 g in 500 ml water) with the addition of ∼4.9 ml of 85% H_3_PO_4_ to give pH 2. Purification and collection of product peaks were achieved using the same column at a flow rate of 0.5 ml/min and monitoring elution of products on the diode array detector. After collection, the phosphate buffer was removed by addition of water and extraction on an Oasis cartridge with final elution using 70:30 acetonitrile/water.

The NAC-methyl ester adducts with 13-oxo-ODE methyl ester was isolated by RP-HPLC using a Waters Symmetry 5 μ C18 column (25 × 0.46 cm) and an isocratic reversed-phase solvent of acetonitrile/water/glacial acetic acid 60:40:0.01 by volume and further purified by straight-phase HPLC, with the two major diastereomers chromatographing as a single peak in both solvent systems. On NP-HPLC, the NAC-Me adducts were run on a Thomson Advantage 5 μ silica column (25 × 0.46 cm) with a solvent of hexane/IPA (90:10 by volume) at a flow rate of 0.5 ml/min (retention time 19 min).

The 13-oxo-ODE-BME adducts were separated by RP-HPLC using the same column and conditions as for the NAC-methyl ester adducts. As on RP-HPLC, the BME adducts were well resolved on NP-HPLC using an Apollo silica 5μ column (25 × 0.46 cm) and a solvent of hexane/isopropanol/glacial acetic acid (100:5:0.02 by volume) and a flow rate of 1 ml min (retention times 7.2 and 9.0 min).

### LC-MS analyses

High-resolution LC-MS of the thiol adducts of 13-oxo-ODE used a Thermo Q Exactive HF Hybrid Quadrupole-Orbitrap (Thermo Fisher Scientific). RP-HPLC-MS analysis was performed with electrospray ionization in positive mode and with atmospheric pressure chemical ionization (APCI) in negative mode. An Agilent C18 5 μ column (15 × 0.2 cm) was eluted isocratically with acetonitrile/water/glacial acetic acid (typically 50:50:0.01 by volume although adjusted for different analytes) at a flow rate of 0.3 ml/min. The electrospray voltage was set at 4.5 kV; sheath and auxiliary gas at 40 and 10 respectively; capillary temperature at 320 °C; the aux gas temperature at 350 °C; S lens RF amplitude 60. Full scan MS was performed over a range of 100 to 650 m/z at a resolving power of 30,000. The in-source CID was 10.0 eV for precursor scan and MS^2^ scans and 40 eV for pseudo-MS^3^ experiments incorporating in-source fragmentation (see Tables S# and S#) Negative ion APCI source parameters were optimized as follows: Corona discharge current 10 μA; sheath and auxiliary gas at 40 and 10 respectively; capillary temperature at 320 °C; APCI heater temperature 350 °C; S lens RF amplitude 60.

For the acquisition of genuine MS^3^ spectra, LC-MS analysis was also performed using an Orbitrap XL hybrid linear ion trap-orbitrap high-resolution mass spectrometer (Thermo) interfaced with a Waters Acquity UPLC system (Waters Corp). High-resolution mass spectra were acquired in positive ion mode over a precursor ion range of *m/z* 165 to 650 at a resolving power of 30,000 using the following ESI source parameters: spray voltage 5 kV; capillary temperature 275 °C; HESI temperature 300 °C; tube lens 150 V; N_2_ sheath gas 40; N_2_ auxiliary gas 10; and source fragmentation of 5 V. MS^3^ experiments were performed using the isolation and activation parameters in Table S#.

### NMR analyses

H NMR and ^1^H,^1^H COSY NMR experiments were acquired using a 14.0 T Bruker magnet equipped with a Bruker AV-III console operating at 600.13 MHz. All spectra were acquired in 3 mm NMR tubes using a Bruker 5 mm TCI cryogenically cooled NMR probe. Chemical shifts were referenced internally to the middle acetonitrile peak at 1.93 ppm in CD_3_CN/D_2_O (60:40 by volume) or benzene-*d*_6_ (7.16 ppm). In samples dissolved in CD_3_CN/D_2_O mixtures, occasionally the auto-shim program failed to lock on the D_2_O signal and the samples had to be manually shimmed. Although the issue was not clearly resolved, this appeared less likely to occur with samples freshly mixed and transferred to the 3 mm tubes for prompt NMR analysis. Possibly there is a phase-gradient issue with the two solvents. Remixing of problematic samples typically solved this issue and the auto-shim worked normally.

## Data availability

All data will be made available upon reasonable request.

## Supporting information

This article contains supporting information (14, 39, 40, 41).

## Conflict of interest

The authors declare that they have no conflicts of interest with the contents of this article.
