# Supplementary material for: The Michael addition of thiols to 13-oxo-octadecadienoate (13-oxo-ODE) with implications for LC-MS analysis of glutathione conjugation
Source: J Biol Chem. 2024 Apr 16;300(5):107293. doi: 10.1016/j.jbc.2024.107293 (PMC11109300; doi:10.1016/j.jbc.2024.107293)
Supplement: Supporting Information [file mmc1.pdf]

Supporting Information

The Michael addition of thiols to 13-oxo-octadecadienoate (13-oxo-ODE) with implications for LC-MS analysis of glutathione conjugation

William E. Boeglin, Donald F. Stec, Saori Noguchi, M. Wade Calcutt, and Alan R. Brash

Contents:

Figure S1: Comparison of consecutive injections of the same 13-oxo-ODE-GSH sample, same column, same amount injected, with solvents of (A) CH_3CN/H_2O/glacial acetic acid (35:65:0.01 by volume) and (B) 35:65 CH_3CN/50 mM potassium phosphate, pH 2.

Figure S2: RP-HPLC analysis of *unreacted* substrate after reaction of 13-oxo-ODE with glutathione

Figure S3: ^{1}H-NMR spectrum and COSY analysis of P3, a major 1,6 adduct of 13-oxo-ODE-GSH (600 MHz, in CD_3CN/D_2O, 60:40 by volume)

Figure S4: Precise alignment of the proton NMR spectra of the two major 13-oxo-ODE-GSH conjugates (P3 and P4, cf. Fig. 2) illustrates very slight differences in chemical shift

Figure S5: ^{1}H-NMR spectrum and COSY analysis of 13-oxo-ODE-GSH adduct P1 (from Fig. 2 main text), (600 MHz, in CD_3CN/D_2O, 60:40 by volume)

Figure S6: ^{1}H-NMR spectrum and COSY analysis of 13-oxo-ODE-GSH adduct P8 (from Fig. 2 main text), (600 MHz, in CD_3CN/D_2O, 60:40 by volume)

Figure S7: ^{1}H-NMR spectrum and COSY analysis of the major NAC-methyl ester adduct with 13-oxo-ODE-methyl ester (600 MHz, in d_6-benzene)

Figure S8: Positive ion ESI with MS^3 mass spectra (m/z 602 $\rightarrow$ 473 $\rightarrow$ MS^3) of four species of 13-oxo-ODE-GSH adducts

Figure S9: Negative ion APCI with mass spectra of three species of 13-oxo-ODE-GSH adduct

Supporting Information text

Proton NMR spectral data

SI References

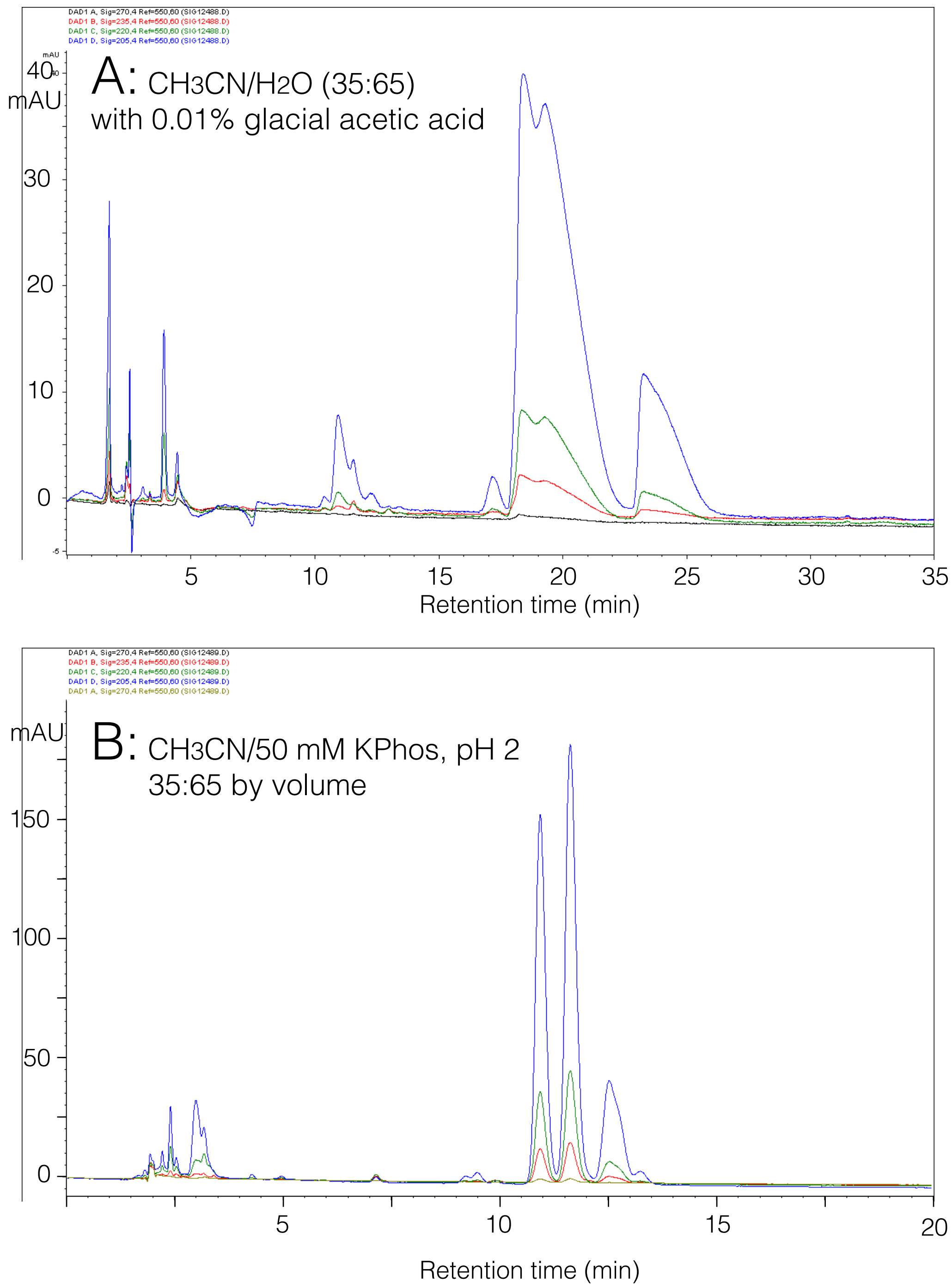

Figure S1: Comparison of consecutive injections of the same 13-oxo-ODE-GSH sample, same column, same amount injected, with solvents of (A) CH_3CN/H_2O/glacial acetic acid (35:65:0.01 by volume) and (B) 35:65 CH_3CN/50 mM potassium phosphate, pH 2

An equal aliquot of 13-oxo-ODE-GSH conjugates formed by equine GST was injected on a Waters 5 μ C18 Symmetry column (25 x 0.46 cm) eluted at 1 ml/min with (A) CH_3CN/H_2O/glacial acetic acid (35:65:0.01, by volume) **or** (B) CH_3CN/50 mM KH_2PO_4 adjusted to pH 2 with 85% H_3PO_4 (35:65, by volume). In addition to the differences in peak shape and resolution, note the difference in absorbance readings (mAU) on the 13-oxo-ODE-GSH conjugates. The UV signals are 205 nm (blue), 220 nm (green), 235 nm (red), and 270 nm (black). The sample was prepared using equine GST transformation of 13-oxo-ODE.

SI Fig. S2

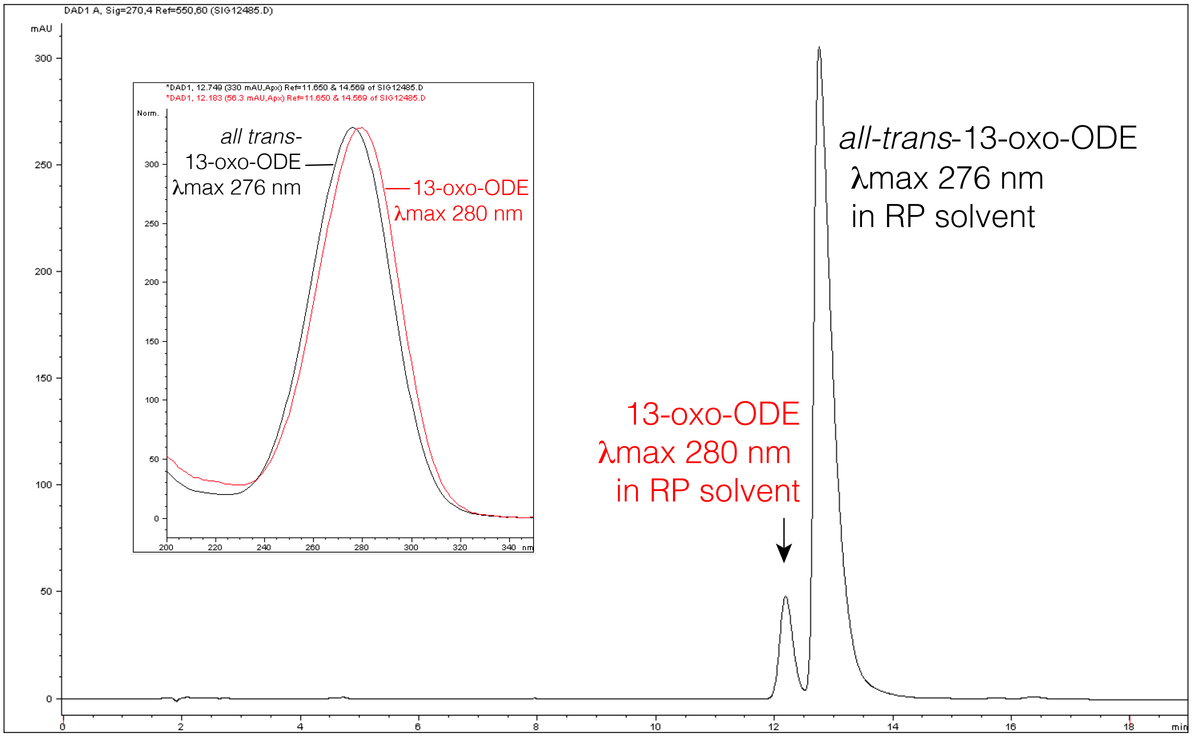

Figure S2: RP-HPLC analysis of unreacted substrate after reaction of 13-oxo-ODE with glutathione

The unreacted substrate was analyzed by RP-HPLC using a Waters 5 μ Symmetry C18 column (25 x 0.46 cm) with a solvent of $CH_3CN/H_2O/HAc$ (70:30:0.01 by volume) at a flow rate of 1 ml/min with UV detection at 270 nm. The earlier eluting 13-oxo-ODE and the all-trans-13-oxoODE isomer are distinguished by the characteristic UV spectra shown in the inset. The example illustrated here was catalyzed by equine GST on 13-oxo-ODE, although the non-enzymic reactions result in similar isomerization of the unreacted substrate.

SI Fig. S3

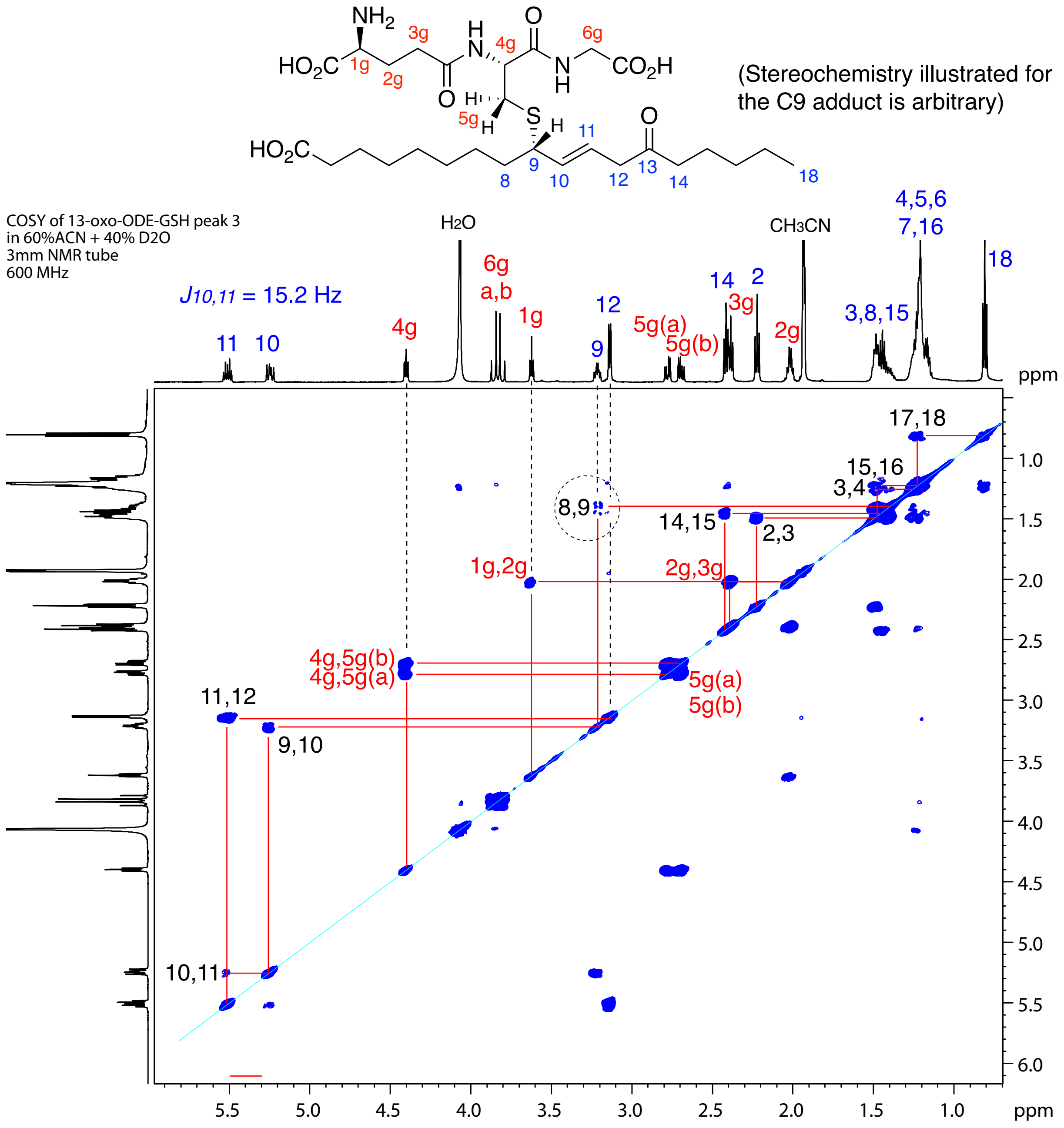

Figure S3: ^{1}H-NMR spectrum and COSY analysis of P3, a major 1,6 adduct of 13-oxo-ODE-GSH (600 MHz, in CD_3CN/D_2O, 60:40 by volume)

In the COSY analysis of isomer, the cross-peak for H8-H9 is weak and the circled copy under H9 at 3.2 ppm was produced in an increased contour level of the COSY

SI Fig. S4

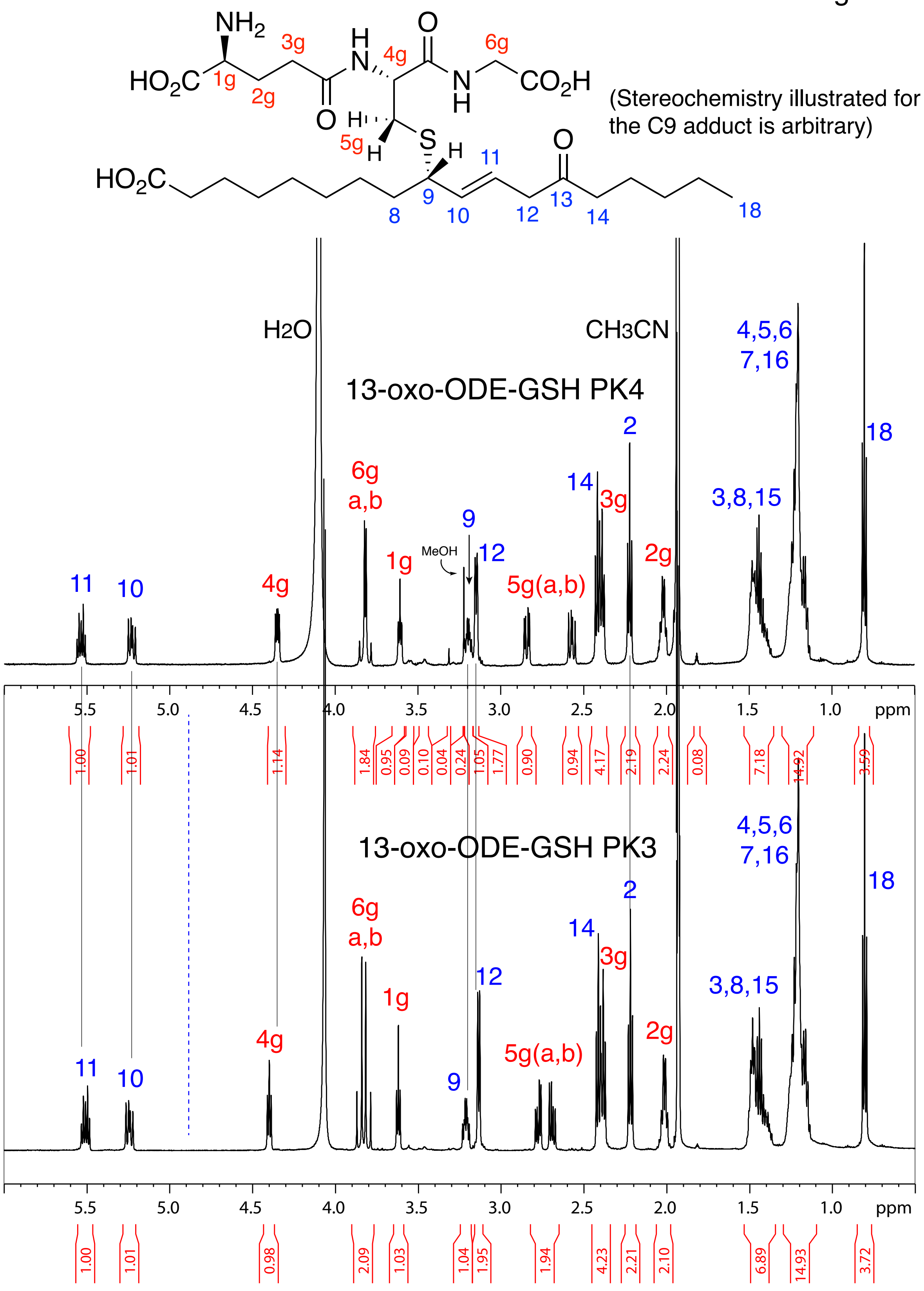

Figure S4: Precise alignment of the proton NMR spectra two major 13-oxo-ODE-GSH conjugates illustrates very slight differences in chemical shift

Vertical lines are added to help view and compare the alignments

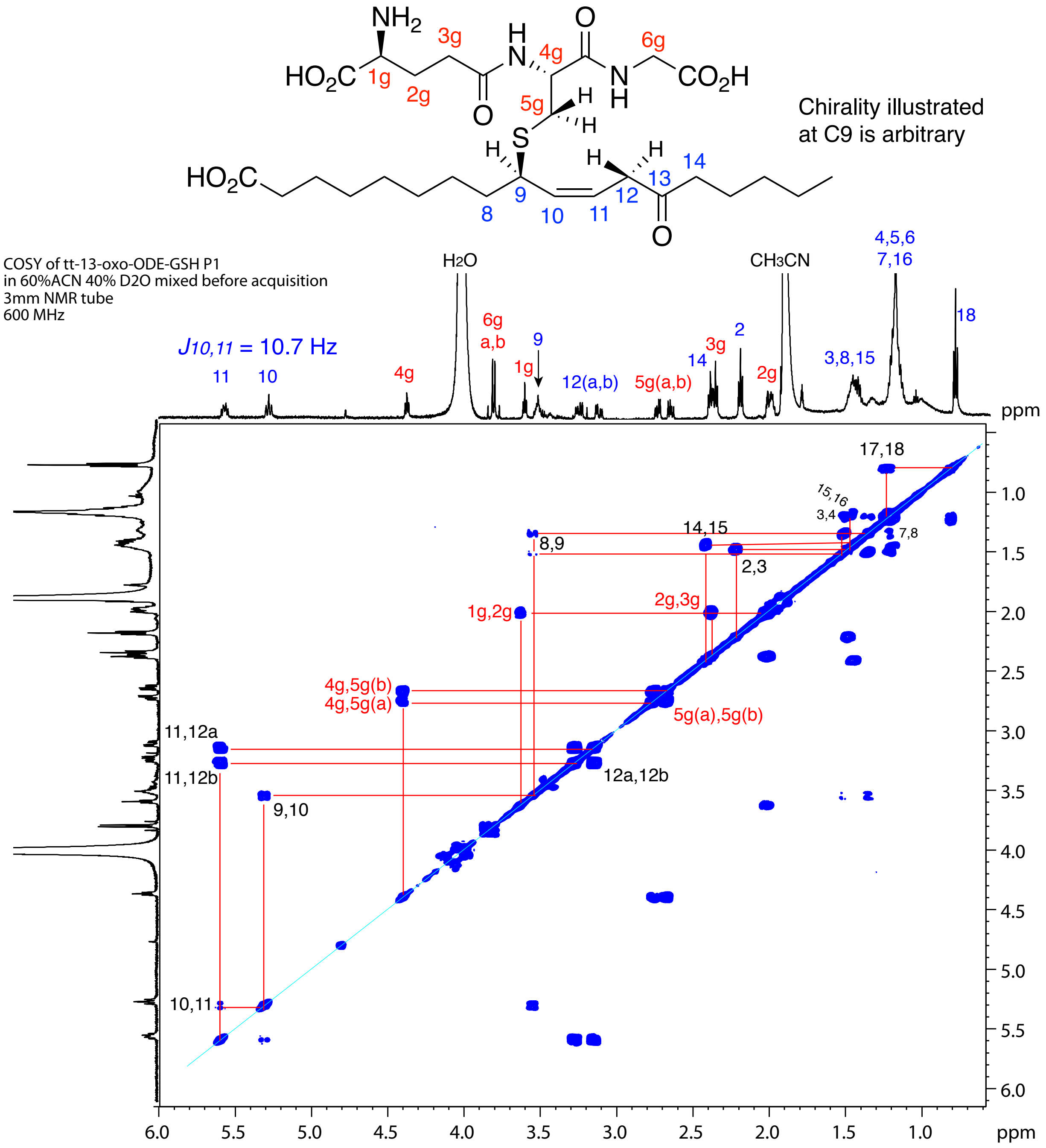

Figure S5: ^{1}H-NMR spectrum and COSY analysis of 13-oxo-ODE-GSH adduct P1 (from Fig. 2 main text), (600 MHz, in CD_3CN/D_2O, 60:40 by volume)

SI Fig. S6

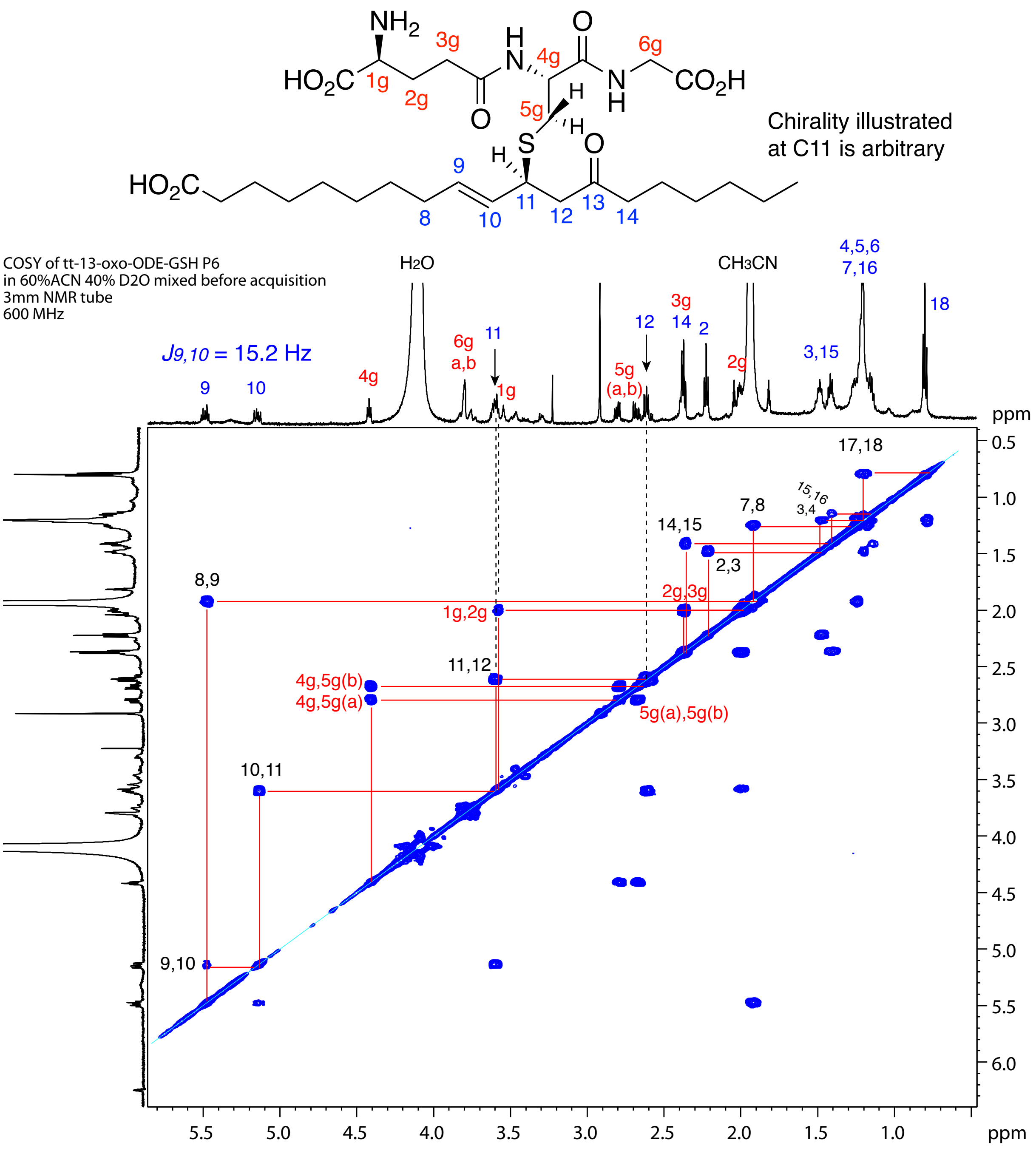

Figure S6: ^{1}H-NMR spectrum and COSY analysis of 13-oxo-ODE-GSH adduct P8 (from Fig. 2 main text), (600 MHz, in CD_3CN/D_2O, 60:40 by volume)

SI Fig. S7

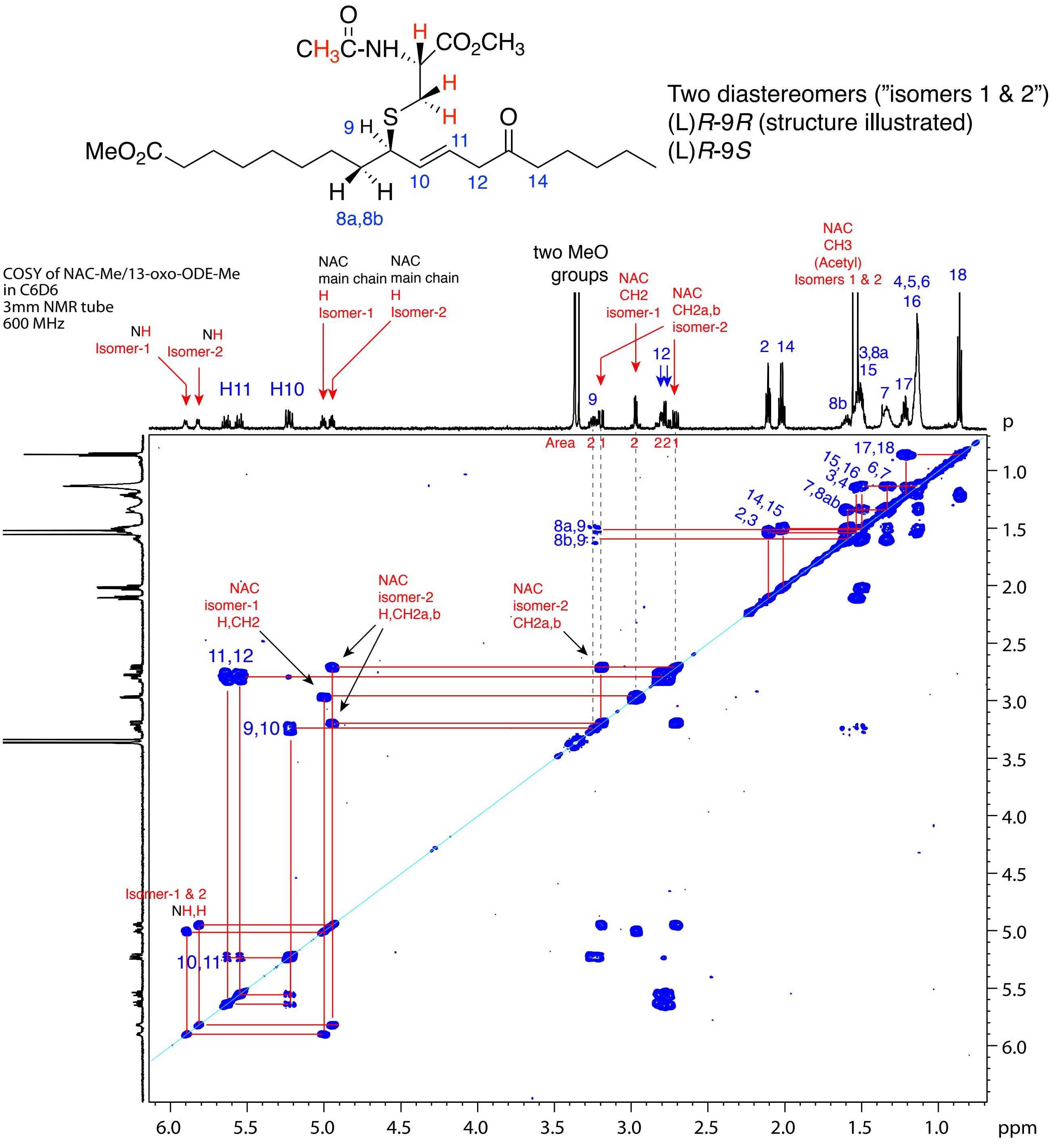

Figure S7: ^{1}H-NMR spectrum and COSY analysis of the major NAC-methyl ester adduct with 13-oxo-ODE-methyl ester (600 MHz, in d6-benzene).

Two equal abundance diastereomers (isomer-1 and isomer-2) are partly resolved in the proton NMR spectrum. (The two co-chromatograph on RP-HPLC and NP-HPLC).

SI Fig. S8

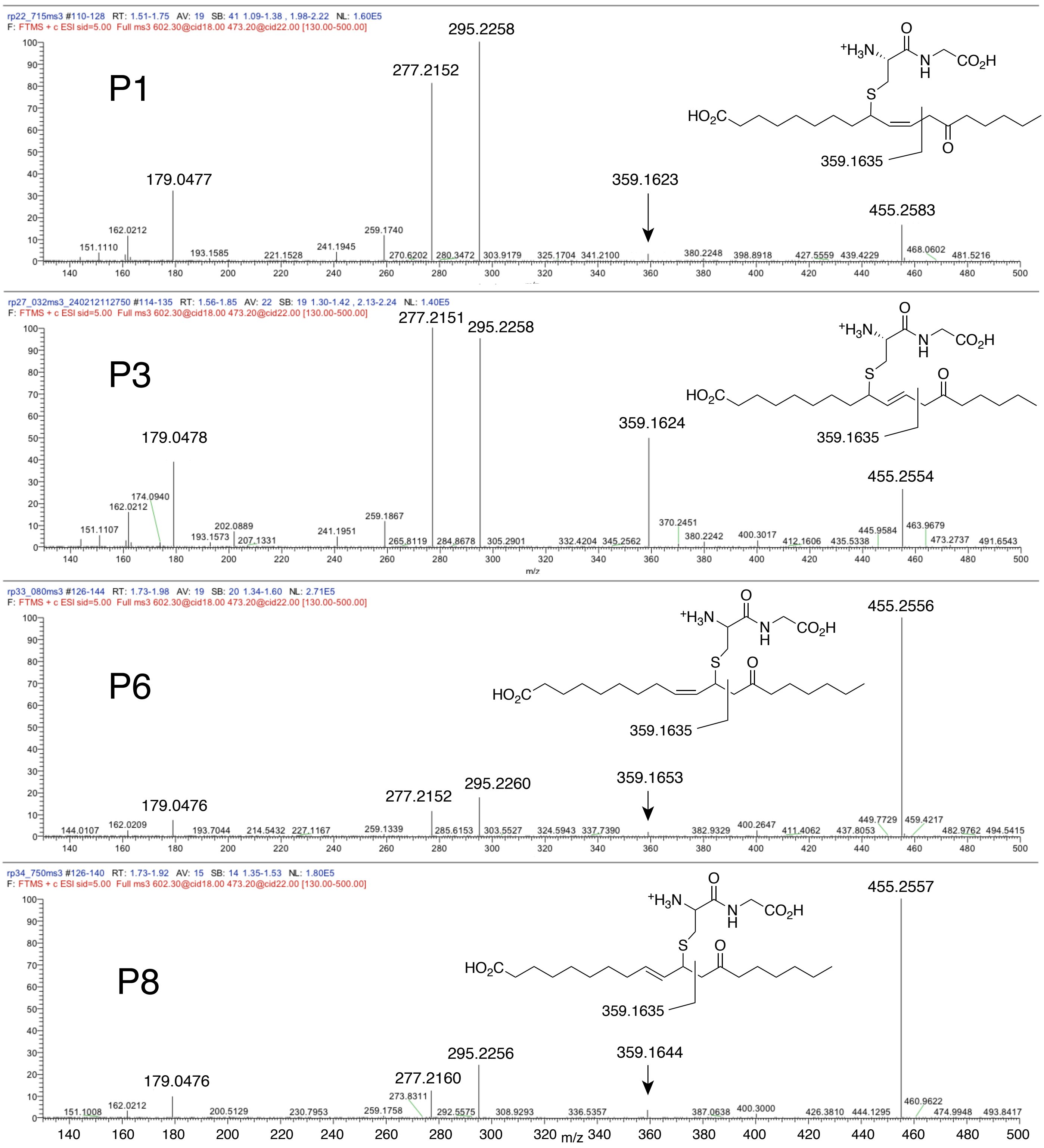

Figure S8: Positive ion ESI with MS3 mass spectra (m/z 602→473→MS3) of four species of 13-oxo-ODE-GSH adducts

The glutathione conjugates represent one each of the four pairs in Figure 2 (main text) with the structures indicated on the mass spectra. MS3 of the m/z 473 ion produces m/z 359 in all four purified isomers.

SI Fig. 9

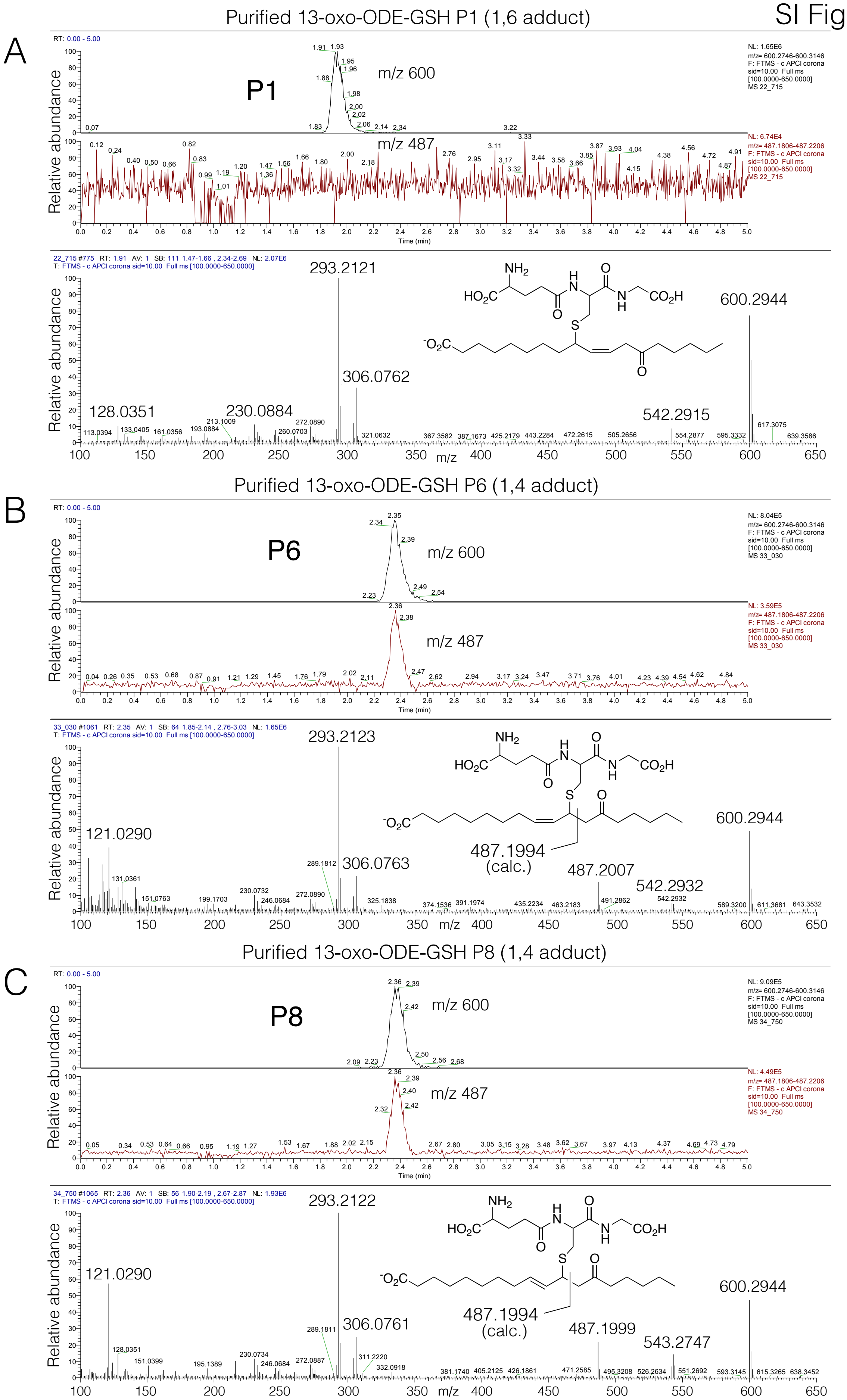

Figure S9: Negative ion APCI with mass spectra of three species of 13-oxo-ODE-GSH adduct: P1, P6, and P8 correspond to the 13-oxo-ODE-GSH adducts in Figure 2 (with P3 and P5 illustrated in Fig. 8, main text). Their structures are indicated on the mass spectra. In these representative spectra, only P6 and P8, the 1,4 addition products with GSH at C11 of 13-oxo-ODE, give an ion at m/z 487.

Comments on the HPLC purification of fatty acid peptidyl adducts

The cysteinyl adducts of 13-oxo-ODE exhibit almost conventional chromatographic behavior when injected in small amounts (less than 5 micrograms on a column with 0.46 cm internal diameter), but injection of larger amounts is associated with detrimental chromatographic performance with progressive peak broadening and earlier elution from the column. This occurs using C18 columns and solvents of acetonitrile/water or methanol/water and acidified with acetic acid or trifluoroacetic acid or with 10 mM ammonium acetate adjusted to pH 5 - 6, the latter a well-recognized solvent for analytical-scale chromatography of cysteinyl leukotrienes (e.g. refs (1, 2)). Furthermore, the expected diastereomeric 13-oxo-ODE-GSH adducts chromatograph within a single broad HPLC peak (Fig. 2A, SI Fig. 1A). In seeking alternative HPLC solvents, we tested a solvent mixture described in the literature of reversed-phase chiral columns (Instruction manual for Chiralpak® AD-RH columns, Chiral Technologies): the aqueous component is 50 mM potassium phosphate adjusted to pH 2 using phosphoric acid, and it takes substantial additions of 85 % H_3PO_4 to bring the solution down to pH 2 (~ 5 ml 85% H_3PO_4 for 500 ml 50 mM KH_2PO_4), indicating strong buffering capacity. Not only does this buffer avoid the problems with peak broadening when 50 - 100 µg amounts are injected on column, unlike the other solvents we tested, it nicely resolves the main 13-oxo-ODE-GSH diastereomers (Fig. 2B, 2C, SI Fig. 1B). Similarly strong phosphate buffer in the pH 6 – 7 range may provide greatly improved chromatography of these polar conjugates and avoid acidic conditions, for example for epoxide conjugates.

Issues with the published NMR data

There are small differences in the chemical shifts of the key protons of the two major 1,6 addition 13-oxo-ODE-GSH diastereomers (illustrated in Supporting Information Fig. S4). When the two isomers are not resolved by HPLC, the small differences in chemical shifts partly merge and blur the individual signals and interfere with clear interpretation; this undoubtedly hampered the original study in which the NMR COSY spectra were obtained on both isomers together (3). On account of overlapping signals from diastereomers, the published data provide no clear or defined structural interpretation. Blackburn et al were correct about the major adduction at C9 of 13-oxo-ODE, although incorrect on the final structure with misplaced double bond (3).

Evidence that P5 and P6 are the 1,4-addition products retaining the 9,10-*cis* double bond

- Mass spectra confirm P5 and P6 are 13-oxo-ODE-GSH adducts.
- As for other 1,4 versus 1,6 adducts identified here, the 1,4 adducts are less polar on RP-HPLC, thus compatible with the elution of P5 & P6 after the 1,6 adducts P1-P4.
- Furthermore, P1 & P2 with a *cis* double bond elute before the corresponding *trans* isomers (P3 & P4), compatible with the same order of elution for the 1,4 adducts P5 & P6 with a 9,10-*cis* double bond eluting before their 9,10-*trans* isomers P7 and P8.
- The products P5 and P6 are only evident in the RP-HPLC profile from 9*cis*,11-*trans*-13-oxo-ODE (Fig. 2B) and not from *all-trans*-13-oxo-ODE (Fig. 2B).
- Equine GST forms P5 in higher abundance than P6, or P7 & P8, compatible with a preferential formation of one diastereomer that retains the 9,10-*cis* double bond.
- In negative ion APCI spectra, P5 & P6 share with the 1,4-adducts P7 & P8 the formation of m/z 487 ion representing C1-C11 plus the GSH tripeptide.
- By all of the above and by a process of elimination, P5 and P6 are the 1,4-adduction products at C11 of 13-oxo-ODE and retaining the 9,10-*cis* double bond.

Proton NMR spectra of the two BME adducts:

Whereas the non-enzymic addition of GSH or N-acetyl-cysteine occurs very predominantly at C9 of 13-oxo-ODE, the reactions with BME showed adduction occurred about equally at the 9- and 11-carbons. Also, they could be analyzed on NMR in $CDCl_3$ or even d_6-benzene, quite an advantage over the more polar NMR solvents with interfering peaks of water, methanol or acetonitrile. In d_6-benzene, the signal from any remaining water is at 0.5 ppm, well removed from signals of these analytes. The availability of both the 1,4 and 1,6 adducts with BME makes the respective assignments all the more secure.

Analysis of the proton NMR spectrum of RP1-NP2, the more polar 1,6 adduct at C9 of 13-oxo-ODE, is given in the main text. The less polar isomer, RP2-NP1, showed clear signals indicating 1,4 adduction of BME at C11 of 13-oxo-ODE (Fig. 5B). The CH_2 protons at C12 have different chemical shifts due to their chiral neighbor, and as a consequence couple to each other in addition to H11, a double triplet at 4.38 ppm and the site of the thiol adduction. When compared to the chemical shift of H9 at the site of thiol adduction in the more polar adduct, H11 is deshielded and 1.2 ppm further downfield on account of being α to the 10,11 double bond and β to the 13-ketone. H11 couples to the more upfield of the two double bond signals, H10 at 5.20 ppm. Based on coupling constants, the 9,10 double bond retains its *cis* configuration ($J_{9,10}$ = 10.5 Hz). From there, the coupling goes upfield to the two H8 protons at 2.05 and 2.17 ppm, which are split, apparently due to "seeing" or being sensitivity to the chirality at C11. (Another example of this relatively remote splitting of a CH_2 that is not vicinal to a chiral center is illustrated in the proton spectrum of 12-HETE (12-hydroxy-eicosatetraenoic acid), in which the methylene protons at C7 have separate chemical shifts, in that case attributed to a cyclic conformation of the molecule in aprotic solvents with hydrogen bonding between the C12 hydroxyl and a carboxylate oxygen (4)). Altogether, these couplings and including the *cis*-9,10 double bond establish the structure of BME adducted to C11 of the less polar adduct.

NMR of 13-oxo-ODE adducts

The full spectrum for the **13-oxo-ODE-GSH P3** in Fig. 2: ^{1}H-NMR Chemical shift δ, multiplicity, number of protons, proton number, and *J*: (600 MHz, CD_3CN/D_2O) δ (ppm) 600 MHz, δ 5.51 (1H, dt, J = 7.3 Hz, 15.0, H11); 5.24 (1H, dd, J = 9.3 Hz, 15.0, H10); 4.40 (1H, t, J 8.0, 4g); 3.85, 1H, d, J17.5, 6g-a); 3.80 (1H, d, J17.5, 6g-b); 3.62 (1H, t, J 6.4, 1g); 3.21 (1H, m, H9); 3.13 (2H, d, J 7.1, H12); 2.77 (1H, dd, J 5.9, 13.9, 5g-a); 2.69 (1H, dd, J 7.6, 13.9, 5g-b); 2.41 (2H, t, J 7.4, H14); 2.38 (2H, t, J 7.4, H2); 2.22 (2H, t, J, H2); 2.01 (2H, q, J7.6, 2g); 1.36 – 1.52 (6H, m, H3, H8, H15); 1.3 -1.28 (m, 10H, H4 - H7, 16); 0.80 (3H, t, 7.1, H18).

The full spectrum for the **13-oxo-ODE-GSH P4**, ^{1}H-NMR Chemical shift δ, multiplicity, number of protons, proton number, and *J*: (600 MHz, CD_3CN/D_2O) δ (ppm) 600 MHz, δ 5.54 (1H, dt, J = 7.5 Hz, 15.2, H11); 5.23 (1H, dd, J = 9.7 Hz, 15.3, H10); 4.35 (1H, dd, J, 4g); 3.81, 2H, d, J 8.3, 6g-ab); 3.61 (1H, t, J 6.4, 1g); 3.20 (1H, m, H9); 3.15 (2H, d, J 6.8, H12); 2.84 (1H, dd, J 4.9, 14.1, 5g-a); 2.57 (1H, dd, J 9.5, 14.1, 5g-b); 2.41 (2H, t, J 7.4, H14); 2.39 (2H, t, J 7.5, 3g); 2.22 (2H, t, J 7.4, H2); 2.01 (2H, q, J 7.0, 2g); 1.35 – 1.51 (6H, m, H3, H8, H15); 1.13 -1.27 (m, 10H, H4, 5, 6, 7, 16); 0.80 (3H, t, 7.2, H18).

13-oxo-ODE-Me-NAC-Me

The two isomers were recorded together in d_6-benzene; assignments are on the COSY (SI Fig. S5): 5.90 (1H, br.d J ~8, Isomer-1 NH); 5.82 (1H, br. d, J ~ 7, Isomer-2 NH); 5.63 (1H, dt, J 7, 15.3, H11(a)); 5.55 (1H, dt, J 7, 15.3, H11-b); 5.225 (2H, dd, j, 9.6, 15.3, two superimposed H10); 5.00 (1H, m, NAC-main chain-H, isomer-1); 4.95 (1H, m, NAC-main chain-H, isomer-2); 3.36 and 3.33 (both 3H, s, fatty acid $CO_2\underline{CH_3}$ and NAC-$CO_2\underline{CH_3}$); 3.24 (2H, m, H9); 3.19 (1H, dd, J 4.7, 13.6, NAC-CH_2-a isomer-2); 2.96 (2H, dd, J 4, 5, NAC-CH2-isomer-1); 2.79 – 2.84 (2H, m, H12); 2.76 (2H, dd, J 7, 17, H12 second isomer); 2.11 (4H, dt, (almost superimposed t), H2 both isomers); 2.02 (3H, dt, superimposed H14 both isomers); 1.59 (2H, m, H8a both isomers); 1.55 and 1.51 (6H, two singlets, NAC- acetyl- CH_3 two isomers); 1.47 – 1.56 (m, H4, H8ab, H15); 1.33 (H4, m, H7 two isomers); 1.21 (4H, m, H17 both somers); 1.10 – 1.17 (m, H4-6, H16); 0.85 (6H, t, H18 both isomers).

13-oxo-ODE-BME RP2-NP1, less polar, 1,4 adduct, (600 MHz, C_6D_6) δ (ppm) 600 MHz, δ 5.39 (1H, dt, J 10.6, H11); 5.18 (1H, t/dd, J 10.6, H10); 4.38 (1H, dt, J 7.0, 10.6, H11); 3.59 (2H, m, BME-CH_2[OH]); 3.359 (3H, s, $CO_2\underline{CH_3}$); 2.59 (1H, dt/ddd, J 6, 13.8, BME-$\underline{CH_2}$-a[SH], H12); 2.49 (1H, dt, J 6.2, 13.8, BME-$\underline{CH_2}$-b[SH]); 2.38 (1H, dd, J 6.6, 16.7, H12a); 2.28 (1H, dd, J 7.4, 16.7, H12b); 2.17 (1H, m, H8a); 2.12 (2H, t, J 7.5, H2); 2.05 (2H, m, BME-<u>OH</u>, H8b); 1.94 (2H, m, H14); 1.55 (2H, m, H3); 1.45 (2H, quin, J 7.6); 1.27 (2H, m, H7); 1.14 - 1.22 (6H, m, H4-6, H17); 1.08 (2H, m, H16); 0.83 (3H, t, J 7.3, H18).

13-oxo-ODE-BME RP1-NP2, more polar, 1,6 adduct, (600 MHz, C_6D_6) δ (ppm) 600 MHz, δ 5.40 (1H, dt, J 6.9, 15.3, H11); 5.18 (1H, dd, J 9.5, 15.3, H10); 3.59 (2H, m, BME-CH_2[OH]); 3.366 (3H, s, $CO_2\underline{CH_3}$); 3.17 (1H, m/ddd, H9); 2.69 (3H, m, BME-$\underline{CH_2}$-a[SH], H12); 2.49 (1H, dt, J 6.9, 13.7, BME-$\underline{CH_2}$-b[SH]); 2.11 (3H, t & m, J 7.5, H2); 1.94 (2H, t, J 7.4, H14); 1.43-1.62 (6H, m, H3, H15, H8); 1.32 (2H, m, H7); 1.19 (2H, m, H17); 1.08-1.16 (m, H4-6, H7, H16); 0.85 (3H, t, J 7.3, H18).

References for Supporting Information

1. Williams, J. D., Czop, J. K., and Austen, K. F. (1984) Release of leukotrienes by human monocytes on stimulation of their phagocytic receptor for particulate Activators. *J. Immunol.* **132**, 3034-3040
2. Metz, S. A., Hall, M. E., Harper, T. W., and Murphy, R. C. (1982) Rapid extraction of leukotrienes from biologic fluids and quantitation by high-performance liquid-chromatography. *J. Chromatog.* **233**, 193-201
3. Blackburn, M. L., Ketterer, B., Meyer, D. J., Juett, A. M., and Bull, A. W. (1997) Characterization of the enzymatic and nonenzymatic reaction of 13-oxooctadecadienoic acid with glutathione. *Chem. Res.Toxicol.* **10**, 1364-1371
4. Bernart, M. W., and Gerwick, W. H. (1994) Eicosanoids from the tropical red alga *Murrayella periclados*. *Phytochemistry* **36**, 1233-1240
